# Phonological acquisition depends on the timing of speech sounds: Deconvolution EEG modeling across the first five years

**DOI:** 10.1126/sciadv.adh2560

**Published:** 2023-11-01

**Authors:** Katharina H. Menn, Claudia Männel, Lars Meyer

**Affiliations:** ^1^ Research Group Language Cycles, Max Planck Institute for Human Cognitive and Brain Sciences, Stephanstr. 1a, 04103 Leipzig, Germany.; ^2^ Department of Neuropsychology, Max Planck Institute for Human Cognitive and Brain Sciences, Stephanstr. 1a, 04103 Leipzig, Germany.; ^3^ International Max Planck Research School on Neuroscience of Communication: Function, Structure, and Plasticity, Stephanstr 1a, 04103 Leipzig, Germany.; ^4^ Department of Audiology and Phoniatrics, Charité – Universitätsmedizin Berlin, Augustenburger Platz 1, 13353 Berlin, Germany.; ^5^ Clinic for Phoniatrics and Pedaudiology, University Hospital Münster, Albert-Schweitzer-Campus 1, 48149 Münster, Germany.

## Abstract

The late development of fast brain activity in infancy restricts initial processing abilities to slow information. Nevertheless, infants acquire the short-lived speech sounds of their native language during their first year of life. Here, we trace the early buildup of the infant phoneme inventory with naturalistic electroencephalogram. We apply the recent method of deconvolution modeling to capture the emergence of the feature-based phoneme representation that is known to govern speech processing in the mature brain. Our cross-sectional analysis uncovers a gradual developmental increase in neural responses to native phonemes. Critically, infants appear to acquire those phoneme features first that extend over longer time intervals—thus meeting infants’ slow processing abilities. Shorter-lived phoneme features are added stepwise, with the shortest acquired last. Our study shows that the ontogenetic acceleration of electrophysiology shapes early language acquisition by determining the duration of the acquired units.

## Teaser

Early phonological acquisition depends on the duration of phonological features (i.e., basic building blocks of phonemes).

## INTRODUCTION

During language acquisition, speech processing is rapidly shaped by the native linguistic environment: Newborns can distinguish all phonemes based on acoustics but lose this ability from 6 months of age onwards. Eventually, they no longer hear the acoustic difference between some sounds that are irrelevant for the understanding of their native language (*1*). Seminal work has shown that English-learning 6- to 8-month-old infants can still distinguish individually presented native and non-native phonemes, yet discrimination of non-native phonemes decreases by 10 to 12 months (*2*). In parallel, the ability to discriminate native phonemes improves (*3*–*7*). On the neurobiological level, auditory association cortex represents phonemes that have been acquired as bundles of so-called phonological features (*8*). Each phoneme in a speaker’s native inventory corresponds to a unique combination of features. These feature-level representations are invoked once an acoustic exemplar of the phoneme is present in speech (*9*, *10*). The exact age at which features are acquired is a matter of debate, with some recent reports that infants as young as 3 months may be able to access feature information of speech sounds (*11*).

The rapid time course of feature acquisition seems paradoxical, because infant brains are too slow for detecting and processing individual phonemes. Infant-directed speech serves ∼20 phonemes per second, resulting in an average duration of ∼50 ms (*12*). Yet even at 7.5 months of age, infants fail to dissociate 70-ms-long sounds that are separated by less than ∼75 ms (*13*). We have recently proposed that this slowness is explained by neurobiological immaturity at birth (*14*): Fast electrophysiological activity that is required for phoneme-rate auditory and feature processing (*9*, *15*) only emerges after birth and approaches adult speeds across infancy and childhood (*16*–*18*). In line with this temporal constraint, newborns initially focus on slow prosodic modulations (i.e., long units) of speech, shifting toward smaller (i.e., faster) units only later [e.g., syllables and phonemes (*14*, *19*)].

**Fig. 1.**
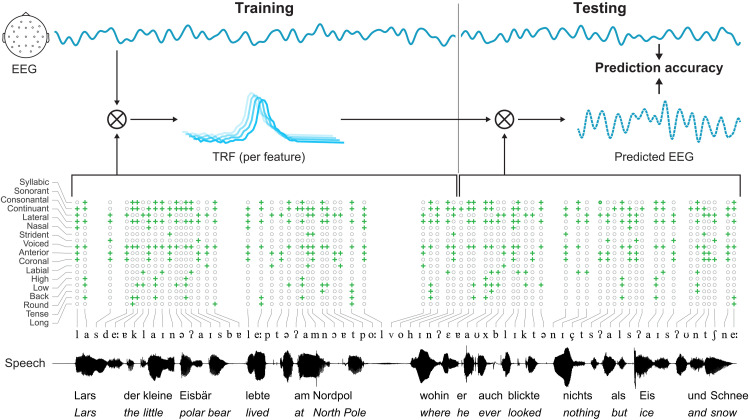
Overview of TRF method. EEG and speech data were split into training and testing data for 10-fold cross-validation. Model parameters were estimated on the basis of the training set. TRFs were then assessed on the previously unseen test set. The correlation between the predicted and observed data (= prediction accuracy) across all folds was used as dependent measure for further statistical analyses.

Individual phonemes in natural speech may be too short for infants’ long temporal processing windows. However, actually not all features alternate at a fast rate. Instead, some features (e.g., voicing and place of articulation) often span sequences of multiple subsequent phonemes (Fig. 1). As a result, features alternate at a much slower rate. In this study, we show that when this rate is slow enough, infants’ electroencephalogram (EEG) clearly indicates sensitivity to features. This suggests that feature timing allows infants to bootstrap into phoneme acquisition in spite of neurobiological slowness. We recorded the EEG from children between 3 months and 4.5 years of age who listened to translation-equivalent stories in their native language (German) and a non-native language (French). French and German largely overlap in their phoneme inventories (*20*, *21*) and children learning either language show similar learning trajectories for individual phonemes (*22*, *23*). Most phonological features are relevant in both languages. One major difference in phonological features between German and French is the [long] feature, which only distinguishes speech sounds in German but not in French. Furthermore, the [nasal] feature is more relevant in French compared to German. In addition, French does not allow for diphtongs. However, the exact acoustic realizations of phonological features differ between the two languages [e.g., (*24*) for voicing], and native speakers of the two languages weigh acoustic cues differently. In addition, there are some prosodic differences between the two languages, which are most evident in their rhythmic structure (*25*, *26*). German is stress timed, meaning that it has roughly equal intervals between stressed syllables, whereas French is syllable timed, that is, syllables have an approximately equal duration. German marks stress on the word level, and the position of the stressed syllable can vary between words (*27*). In contrast, stress is never contrastive on the word level in French. From the EEG collected during story listening, we estimated categorical processing of phonemes from the prediction accuracy of cross-validated feature-based EEG deconvolution models (temporal response functions (TRFs)]. We hypothesized that the prediction accuracy would increase cross sectionally for the native but not the non-native language in line with perceptual narrowing accounts of phonological acquisition (*1*, *2*). Regarding the acquisition of individual features in the native language, we expected that the cross-sectional increase in TRF prediction accuracy for any feature could be predicted by the average duration of this particular feature in child-directed speech even after correcting for the frequency of occurrence of each feature.

## RESULTS

### Native specificity of phonological processing

**Fig. 2.**
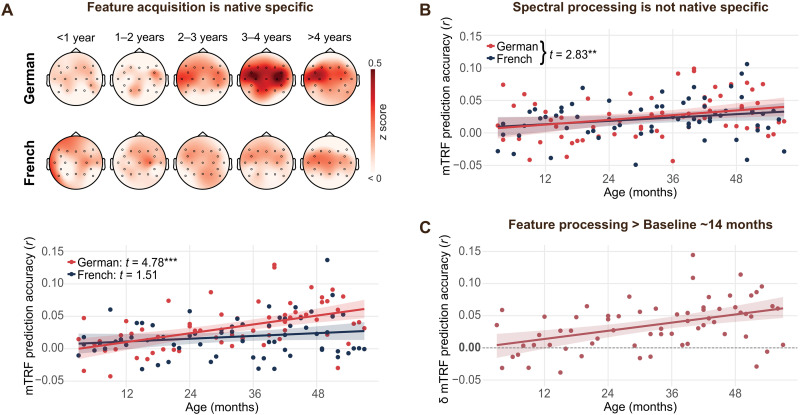
Overview of overall feature acquisition results. (**A**) Feature acquisition is native specific. Top panel depicts topography of the average z-transformed prediction accuracy per year of age for the native language (German) and the non-native language (French). Lower panel shows regression analysis for prediction accuracy for electrode FC5 predicted by participant age for the two language conditions (*N* = 66). Prediction accuracy significantly increases with age for the native (*t* = 4.78, *P* < 0.001) but not for the non-native language (*t* = 1.51, *P* = 0.133). (**B**) The prediction accuracy for spectrogram processing significantly increases with age [*t*(64) = 2.83, *P* = 0.006]. The interaction between age and language condition was not significant [*t*(64) = 0.45, *P* = 0.656], providing no evidence that the increase of acoustic processing is language specific. *N* = 66. (**C**) The difference in prediction accuracy between German and a statistical baseline significantly exceeds zero after 14 months of age. Shaded area depicts the 95% CI. *N* = 66.

To establish consistency with previous literature, we first compared phonological processing in the native language to the non-native language. Our dependent measure was the prediction accuracy of the EEG deconvolution model. High prediction accuracy indicates reliable neural responses to phonological features across the children’s story, as is expected once phonological representations have been formed (*15*). In line with established findings, our mixed-effects model analysis showed a significant interaction between language condition and age, *t*(64) = 2.34, *P* = 0.023 (Fig. 2A). Follow-up analyses showed that the increase of prediction accuracy with age was specific to the native language [*t*(64) = 4.78, *P < *0.001; non-native: *t*(64) = 1.51, *P* = 0.133], indicating an increase in sensitivity to native phoneme features. To assess the age at which native and non-native feature processing diverge, we assessed the fitted confidence interval (CI) for the age trajectory of the difference in prediction accuracy between the native and the non-native conditions. The CI initially includes 0 and exceeds 0 at 28 months of age. This indicates that, in the beginning, there are no differences in prediction accuracy between the two languages in our sample, suggesting that infants initially process the features of both languages similarly and that accuracy for the native language improves with increasing familiarity with their native language. In contrast, we found no evidence for a native specificity of general acoustic processing (Fig. 2B): Mixed-effects models using the spectrogram as an alternative predictor showed no interaction between language condition and age, *t*(64) = 0.45, *P* = 0.656, but only a main effect of age, *t*(64) = 2.83, *P* = 0.006. Our acoustic predictor in this model consisted of logarithmically spaced channels of the full spectrogram from 250 to 8000 Hz, which thus includes the spectrotemporal information relevant for phoneme identification as well as the fundamental frequency. Our results thus indicate the emergence of native phonological representations and a general enhancement in acoustic processing with increasing age.

Control analyses including infant sex showed no significant three-way interaction between age, language, and sex in our sample, *t*(62) = −0.35, *P* = 0.728. Given that sex was not perfectly balanced in our sample, we further used a bootstrapping approach with 1000 sex-balanced bootstrap resamples to estimate the 95% CI for any interaction with sex. The resulting CIs for the *t* values of the interaction terms were all found to include 0 for the model including features (CI age × sex: −1.65 to 2.53; age × language × sex: −2.32 to 1.32) and the model including the spectrogram as dependent variable (CI age × sex: −2.41 to 1.61; age × language × sex: −1.96 to 2.7), suggesting that the interactions are not statistically significant at the 0.05 level. Consequently, our findings indicate no compelling evidence of a moderating effect of sex on the developmental trajectory of phonological feature acquisition. It should be noted, however, that inclusion of age led to an improvement of overall model fit in 28% of the bootstrapping samples for the feature-based model, the interaction between age and sex was only significant for 11% of the samples, and the three-way interaction between age, language, and sex was only significant for 5% of the samples. For control analyses showing that our results cannot be explained by advances in lexico-semantic or syntactic knowledge, see Supplementary text.

### Comparison of feature processing against baseline

To assess at which age children show reliable categorical processing of features from continuous speech above chance, we compared the age trajectory of phonological processing in the native language to age-related changes in prediction accuracy in a statistical permutation baseline. The permutation baseline was created by pairing the EEG data with a randomly time-shifted version of the acoustic predictors for each fold of the cross-validation procedure. This can serve as a statistical baseline by disrupting the true relationship between acoustic predictors and EEG and thus represents the null hypothesis of no systematic relationship between predictor and response. Linear regression analyses showed that native phonological processing was significantly higher than baseline, *t*(64) = 7.93, *P < *0.001, and the difference between observed and baseline prediction accuracy significantly increased with age, *t*(64) = 4.18, *P < *0.001 (Fig. 2C). Fitted CIs across the difference between native and baseline measures show that native categorical processing of phonological features significantly deviates from baseline at 14 months. This indicates that infants show significant categorical processing of phonological features from continuous speech from an age of 14 months.

### Relationship of feature acquisition and duration

**Fig. 3.**
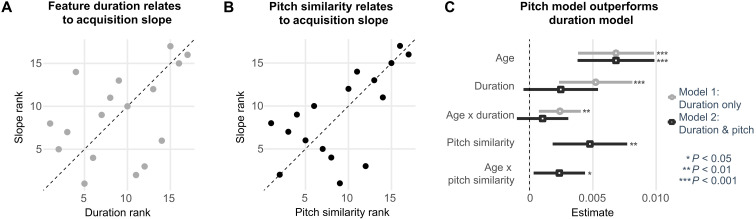
Pitch similarity is a better predictor for feature acquisition than duration. (**A**) Feature duration rank is associated with the feature’s prediction accuracy slope across early childhood. Diagonal line depicts the identity line, which would indicate a perfect match between duration rank and slope rank. (**B**) Pitch similarity rank relates to feature slope across early childhood. (**C**) Comparison of model estimates for the duration model and the full model. For the full model including both pitch similarity and duration rank, only pitch similarity, age, and their interaction are significant predictors for individual feature prediction accuracy.

The average duration of each feature in the native language was measured as the median duration of each feature from feature onset to offset in a recording of natural maternal speech. Duration thus reflects the uninterrupted stretches of multiple phonemes (Table 1). To assess whether duration affects feature acquisition, we conducted a mixed-effects model with age and feature duration rank (from shortest to longest) as predictors. Our results show significant main effects of age, *t* = 4.47, *P < *0.001, and duration rank, *t* = 3.54, *P* = 0.003, on EEG prediction accuracy. The analysis also revealed a significant interaction between age and the duration rank on prediction accuracy, (*t* = 2.83, *P* = 0.005). Here, the age trajectory was steeper for longer features, indicating that infants display categorical sensitivity earlier to those features that extend over longer stretches of speech (Fig. 3A). This effect remained significant after controlling for the overall frequency of occurrence of each feature. This means that even at equal exposure, infants’ sensitivity to a given feature is higher when it tends to extend in time.

**Table 1.** Descriptive statistics for features. Overview of individual feature duration and similarity between the feature and the pitch track. Features were assigned according to Chomsky and Halle (*72*).

Feature name	Median feature duration	Feature duration rank	Pitch similarity rank	Occurence rank
Voiced	228 ms	17	17	17
Continuant	197 ms	16	15	15
Sonorant	178 ms	15	16	16
Low	132 ms	14	5	5
Tense	121 ms	13	10	8
Back	120 ms	12	12	11
Strident	116 ms	11	2	4
Consonantal	116 ms	10	13	14
Long	110 ms	9	6	2
Syllabic	109 ms	8	14	12
Round	109 ms	7	4	3
High	102 ms	6	8	9
Coronal	92 ms	5	9	10
Anterior	90 ms	4	11	13
Nasal	70 ms	3	7	6
Labial	69 ms	2	3	7
Lateral	68 ms	1	1	1

Control analyses assessing possible sex effects on the relationship between feature duration and feature acquisition revealed no significant three-way interaction of sex, age, and language in our sample, *t* = −0.49, *P* = 0.625. We again applied a bootstrapping approach to create sex-balanced samples. The resulting 95% CIs for the *t* values of the three-way interaction between sex, duration rank, and age included 0 (−2.74 to 1.72), providing no evidence of a moderating effect of infant sex on the relationship of feature acquisition and duration at the α = 0.05 level. Inclusion of age improves overall model fit in 38.5% of bootstrapping samples, and the three-way interaction between age, sex, and pitch similarity was significant in 11.3% of bootstrapping samples.

### Exploratory analysis: Prosodic similarity

**Fig. 4.**
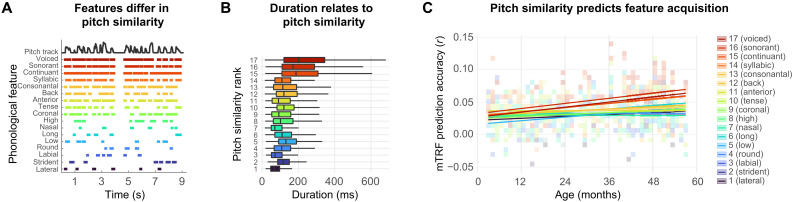
Overview pitch similarity analysis. (**A**) Features differ in their individual resemblance to the pitch track. (**B**) Feature duration associates with the feature’s similarity to the pitch track. Outliers are not plotted here. (**C**) Pitch similarity significantly relates to the developmental trajectory of feature acquisition (*t* = 3.42, *P* < 0.001). Features that share a higher similarity to pitch (higher rank) are acquired faster than features with a lower pitch similarity rank. Shaded tiles depict the average similarity rank in that tile. *N* = 66.

We analyzed whether the acquisition of phonological feature learning may be an extension of infants’ initial focus on prosody in speech processing (*14*). More specifically, we thought that the duration of early-acquired feature-continuous stretches might be similar to the modulation rate of speech prosody (Fig. 4). For this, we first assessed infants’ processing of pitch (fundamental frequency; *F*_0_) in a TRF model using the pitch track as single predictor. In line with previous research, we found significant processing of pitch already in the youngest children (CI: 0.01–0.03; fig. S1), confirming established prosodic processing abilities at an early age (*28*). In addition, we find a significant increase of pitch processing across age, *t*(64) = 2.34, *P* = 0.022. We then quantified the similarity between prosody and each feature as the cross-correlation between the pitch track and the feature predictor. Next, we ranked these from least to most similar (Table 1) and added pitch-similarity rank as a fixed effect to the duration model described above. Including pitch similarity significantly increased the model fit (*P* = 0.001). This model revealed a significant interaction between age and pitch similarity (*t* = 3.42, *P < *0.001; Fig. 4C) as well as significant main effects of pitch similarity (*t* = 3.18, *P* = 0.006) and age (*t* = 4.41, *P < *0.001) on EEG prediction accuracy. This indicates that the more similar the timing of a feature is to the timing of pitch, the steeper its trajectory across age. The interaction between duration and age was not significant in the final model after controlling for pitch similarity (Fig. 3C). All variance inflation factors of the final model were ≤ 3.17, indicating that this result was not caused by multicollinearity. Control analysis revealed no moderating effects of sex on the relationship between pitch similarity and age in our sample, *t* = −0.83, *P* = 0.409. We again used bootstrapping and the resulting 95% CI for the interaction included 0 (CI *t*_age x sex x pitchsimilarity_: −3.32 to 1.34). Inclusion of age improves overall model fit in 66% of bootstrapping samples, the three-way interaction between age, sex, and pitch similarity was significant in 18% of bootstrapping samples. These findings suggest that phonological feature acquisition may be driven by the similarity between prosody and individual phonological features. In other words: At an age when infants’ slow electrophysiology lets them focus on prosody, they also start to process phoneme features that alternate at a comparably slow rate.

## DISCUSSION

In a large cross-sectional sample, we identified the emergence of stable neural activity associated with phonological-feature processing from 14 months of age onwards, using scalp-level EEG deconvolution. This age is later than the previously reported 3 months for feature extraction (*11*), which may be attributed to the use of naturalistic speech in our study, which is more challanging given the rapid transition of phonemes and their increased variability due to co-articulation. Further possible are non-linear slopes for age in the earliest age range, which we could not appropriately model here. We further showed that the development of categorical feature processing is native-specific and not based on a general enhancement of auditory processing, which was found for both the native and the non-native language. This replicates the well-known attunement to native phonological categories during language acquisition (*1*, *2*). We obtained this classical effect under short stimulation with natural continuous speech—that is, at a fraction of the duration needed to assess phonological feature processing in a factorial experiment (*11*, *29*). Moreover, our analyses suggest that the developmental trajectory is a function of the duration of the acoustic counterparts of phonological features in speech: Infants display categorical sensitivity earlier to those features that extend over longer stretches of speech.

Our findings provide evidence that the shaping of language development by electrophysiological maturation extends into the acquisition of fast occurring phonological information, which also progresses from slow to fast phonological features. In infancy and early childhood, slow electrophysiological activity dominates the EEG (*30*), limiting infants’ processing abilities to slow time scales. In contrast, faster electrophysiological activity shows a delayed development across early years (*16*, *31*). The progression of electrophysiological maturation from slow to fast is mirrored in language acquisition (*14*): From birth, newborns can track pitch modulations (*32*) and native prosody may be learned in the womb already (*28*, *33*, *34*), long before infants start building an inventory of native phonology toward the second half of the first year (*1*).

Our exploratory analyses suggest that infants’ ability to use feature-continuous stretches for category acquisition builds on their in-place ability to process prosodic modulations, in particular, frequency modulations at the fundamental frequency (*F*_0_). At a rate of *<*4 Hz (*12*, *35*), these approach the typical frequency of feature alternation (∼4 to 5 Hz for slow features; Table 1), indicating that infants might exploit similar neural mechanisms to extract phonological information as they use for prosody. One could call this possible temporal link between prosodic and phonological acquisition temporal bootstrapping. It has been well established that prosodic modulations are the initial processing objective of infants (*19*, *36*, *37*) and that infants use speech acoustics to infer abstract linguistic knowledge [prosodic bootstrapping (*38*–*41*)]. This is made possible by temporal correspondence between speech acoustics and linguistic units (*42*, *43*). The tracking of prosody may help the acquisition of linguistic meaning (*44*, *45*) by helping to segment and identify linguistically meaningful (e.g., lexico-semantic or syntactic) units in continuous speech. Caregivers appear to support bootstrapping through amplified prosody in infant-directed speech (*46*). In line with the temporal bootstrapping proposal, caregivers prolong phonemes when interacting with infants (*47*, *48*). This may further increase the match between long phonological features and infants’ long neurobiological processing windows. As previous research has indicated a relationship between phonological acquisition and higher linguistic abstraction (*49*), future research should investigate whether the acquisition of phonological features may aid acquisition of higher linguistic knowledge such as vocabulary.

By showing that feature similarity to pitch relates to phonological acquisition, our study provides evidence that early phonological acquisition relies on established prosodic processing abilities (*34*, *50*). Temporal bootstrapping may allow the infant brain to exploit the short-term continuity of phonological features for language acquisition. This helps to solve a major paradox of language acquisition: While infants cannot resolve temporal differences shorter than ∼75 ms (*13*), they do acquire categorical knowledge of phonemes that are even shorter (*12*).

In conclusion, we show that the order of phonological feature acquisition depends on the duration of feature-continuous stretches in speech. The slow electrophysiology of the infant brain and the concomitant tuning to slow prosodic modulations may provide the foundation for the induction of phonological categories from feature-continuous stretches of speech. This helps to explain infants’ astonishing ability to acquire fine-grained linguistic knowledge in spite of their neurobiological limitations.

## MATERIALS AND METHODS

### Participants

Sixty-six children (40 female) between 3 months and 4.5 years were included in the final analysis. Participants’ age was approximately uniformly distributed across the age range. Thirty-one additional infants participated but were excluded for failure to wear EEG cap (*n* = 9), technical failure (*n* = 11), providing insufficient data (*n* = 11; see “EEG analysis” section below). All participants were recruited from the local database of the Max Planck Institute for Human Cognitive and Brain Sciences and were born full-term (*>*37 weeks, *>*2700 g), healthy, and raised in monolingual German environments. None of the children had experienced any exposure to French. Older children were orally informed about the experimental procedure and caregivers were informed both in written and oral form. Caregivers gave written informed consent for their children’s participation in the study. Ethical approval was obtained from the Medical Faculty of the University of Leipzig.

### Materials

The stimuli consisted of three children’s stories in German and in French (Hans de Beer’s *Little Polar Bear and the Husky Pup*, *Little Polar Bear, Take Me Home*, and *Little Polar Bear and the Whales*). Stories were translation equivalent between the two languages and read in a child-directed way by two professional female speakers who were native speakers of German and French, respectively. The recording for each narrative was shortened to 7.5-min duration without compromising content coherence.

For the acoustic models, the spectrogram of the recordings was computed for 16 logarithmically spaced channels between 250 to 8000 Hz (*15*). Phonological annotations (see Table 1) were obtained via forced alignment of orthographic transcript to the acoustic waveform using the WEBMAUS automatic segmentation tool (*51*, *52*). To improve the quality of the alignment, phoneme onsets and labels were manually adjusted by a trained linguist using Praat (*53*). To obtain ecologically valid measures of the average duration of phonological features in German child-directed speech, a feature duration measure was independently obtained by transcribing maternal speech in natural interactions between one mother and her 3-year-old son from the DGD FOLK corpus [∼1 hour of interactions; ∼10,000 individual phonemes produced by the mother (*54*)]. Feature durations were measured as the median duration of each feature from feature onset to offset, thus typically spanning multiple phonemes (Table 1). In case of silence after feature offset, the silent duration was added to the feature duration, as there was no new phonological information interfering with infants’ processing. Features were ranked according to their duration from short to long for subsequent analyses. To control for mere exposure effects, we also ranked the features regarding their overall occurrence in German from least to most frequent.

### Procedure

During the EEG recordings, children were seated on their parent’s lap in an electrically shielded, sound-attenuated booth. They listened to one narrative in German and a different narrative in French. Narratives were presented via speakers and counterbalanced across children. The total experiment lasted 16 min during which EEG was recorded continuously.

### EEG recording and preprocessing

EEG was recorded using a 28-channel EasyCap system (Brain Products GmbH) with Ag/AgCl electrodes arranged according to the 10/10 system. The sampling rate was 500 Hz. Cz served as online reference, and vertical and horizontal electrooculograms were recorded bipolarly. EEG processing was done using the publicly available “eeglab” (*55*) and “fieldtrip” (*56*) toolboxes as well as custom MATLAB code. EEG preprocessing was done automatically using a modified version of the Harvard Automated Preprocessing Pipeline (HAPPE) (*57*). In line with HAPPE, the EEG data were high-pass filtered above 1 Hz with a noncausal finite impulse response filter (order: 1650, −6-dB cutoff: 0.5 Hz) to remove slow drifts that are often artifactual in infant data (*58*). A 45-Hz low-pass filter was applied to remove line noise (order: 332, −6-dB cutoff: 47.5 Hz). Channels were categorized as noisy and removed from preprocessing for any of three reasons: (i) channels that were flat for longer than 30 consecutive seconds; (ii) channels with a Hurst value below 0.7; (iii) channels that exceeded the normed joint probability of the average log power from 1 to 100 Hz by more than 2.75 SD from the mean (mean number of removed channels = 0.83). We applied level 13 wavelet thresholding to remove large artifacts before the previously removed noisy channels were interpolated using spherical splines, which has a high accuracy for low-density EEG data (*59*). Last, the data were re-referenced to the linked mastoids. The 19 channels included in the final analysis were as follows: Fz, F3/4, F7/8, FC5/6, C3/4, Cz, T7/8, CP5/6, Pz, P3/4, and P7/8. We excluded the most posterior channels from the final analysis because the EEG signal was consistently noisy across children.

### EEG analysis

After automatic preprocessing of the continuous EEG data, the TRF analysis was prepared following Jessen *et al.* (*60*): The data were filtered between 1 and 10 Hz before it was segmented into consecutive epochs with a duration of 1 s each. Epochs in which the EEG amplitude in the unfiltered data exceeded a threshold of ±200 μV were rejected automatically. We then combined the EEG data with the acoustic predictors (spectrogram and phonological features) before recombining the EEG epochs, adding 1 s of zeros between nonconsecutive epochs. Children had to contribute at least 350 s (75% of the story) of artifact-free EEG data per language condition to be included in the analysis (M = 434.5 s, SD = 23.6).

To assess the mapping between our acoustic predictors and the EEG data, we used temporal-response functions (TRFs) as implemented in the mTRF toolbox [(*61*); see Fig. 1 for an overview]. For each predictor, a TRF is estimated which best describes the neural response to the respective predictor using regularized linear regression. Individual forward encoding models were computed to predict EEG data from either the acoustic spectrogram or the phonological features separately for the two language conditions (native versus non-native). The spectrogram was added as a continuous predictor, and phonological features were added as a step-function predictor, with steps corresponding to the start- and endpoint of every feature. On the basis of previous research on phonological processing in adults and infants, neural responses were estimated between −150 and 400 ms with respect to feature present samples (*15*, *62*–*65*). The reliability of each model was quantified using 10-fold cross-validation, which quantified the EEG prediction accuracy (Pearson’s *r*) on unseen data while controlling for overfitting. For each iteration, EEG data were split into training (80%), validation (10%), and testing data (10%), which were used as follows: Model parameters were first estimated on the training set. To control for overfitting, the validation set served as a basis for hyperparameter tuning of the regularization parameter, which was performed by means of an exhaustive search of a logarithmic parameter space from 10^−7^ to 10^7^. In the last step, we used the optimized model parameters to predict the previously left-out testing data and quantified the prediction accuracy as the correlation between predicted and observed EEG data. Cross-validation prediction accuracy is high if the infants show a reliable neural response to the features, which can be generalized from the training to the previously unseen test data. We therefore interpret higher TRF prediction accuracy as more stable neural responses and thus an indication of phonological feature acquisition. To assess the statistical significance of the prediction accuracy, we obtained a surrogate distribution of prediction accuracy values under the null hypothesis of no systematic relationship between speech acoustics and EEG data. This surrogate distribution was created by pairing the EEG data with a randomly time-shifted version of the acoustic predictors for each fold of the cross-validation and is referred to as permutation baseline in this manuscript [see (*66*) for a similar approach].

### Statistical analysis

Data were analyzed using mixed-effects models (*67*) in R [v4.1.3 (*68*)] using RStudio [v.2022.7.1.554 (*69*)]. Model significance was assessed using Satterthwaite approximation (*70*). The prediction accuracy for FC5 was used as dependent measure for all analyses, as this was the electrode with the highest overall prediction accuracy in the feature model across children and language conditions. To assess whether processing of phonological features is native specific, we compared the developmental trajectory of the prediction accuracy across all features in German (native) to French (non-native). Age and language (native versus non-native) served as fixed effects and participant as random intercept. For all analyses, age was mean-centered and language was contrast-coded with native as reference level coded as −0.5. In the second analysis, we assessed at which age children show a reliable neural response to phonological features of their native language, by assessing at which age the prediction accuracy across all features in the native language significantly exceeds a statistical baseline. For every child, we subtracted the prediction accuracy computed in the permutation baseline from the actually observed prediction accuracy. Linear regression was used to predict this difference from age. The age at which the prediction accuracy to features is significantly above baseline was determined as the age at which the fitted confidence of the difference between the two measures intervals exceeded zero.

To assess whether the acquisition of individual phonological features relates to their duration in natural speech, the last analysis focused on the predictive accuracy of the 17 individual features in the native language. Features did not occur equally often in our stories, which may have affected TRF model performance. To correct for the possible influence of unequal feature quantities in the training data on the predictive accuracy for individual features, we used the difference between the feature’s observed prediction accuracy and the prediction accuracy computed in the permutation baseline as dependent variable. Age and feature duration rank served as fixed effects, and feature identity and participant were added as random intercepts. Because children are expected to acquire features earlier if they are exposed to them more often, feature occurrence rank was added as an additional control variable to the model.

To estimate the shape of the developmental change, we respectively added logarithmic, quadratic, and cubic terms for age to all of our analyses and compared the respective model fit to the model including only the linear term for age. Because neither of the added terms significantly improved model fit in any analyses, all results are based on linear predictors for age. Previous findings suggest an impact of infant sex on early phonological discrimination abilities (*71*). To assess for a potential effect of sex on our findings, we conducted a control analysis for the main finding including sex as a factor. Given that sex was not perfectly balanced in our sample, we used a bootstrapping technique and constructed a 95% CI for all interaction effects with age from 1000 resamples of a sex-balanced sample of 66 children, thus providing a robust and nonparametric estimation of its uncertainty and allowing for more reliable inferences.

## Supplementary Materials

### This PDF file includes:

Supplementary text Fig. S1 References
