# Supplementary material for: Phonological acquisition depends on the timing of speech sounds: Deconvolution EEG modeling across the first five years
Source: Sci Adv. 2023 Nov 1;9(44):eadh2560. doi: 10.1126/sciadv.adh2560 (PMC10619930; doi:10.1126/sciadv.adh2560)
Supplement: Supplementary file 1 — Supplementary text Fig. S1 References [file sciadv.adh2560_sm.pdf]

Science Advances
AAAS

Supplementary Materials for

Phonological acquisition depends on the timing of speech sounds: Deconvolution EEG modeling across the first five years

Katharina H. Menn *et al.*

Corresponding author: Katharina H. Menn, menn@cbs.mpg.de

Sci. Adv. **9**, eadh2560 (2023)
DOI: 10.1126/sciadv.adh2560

This PDF file includes:

Supplementary text
Fig. S1
References

Supplementary text

Previous research has indicated that higher-order linguistic processing can affect phonological processing in infants' already (49), which means that the increases in linguistic knowledge in older children may have impacted the trajectory feature acquisition described in this manuscript. To control for the increase of higher linguistic knowledge as children age, we have included a predictor for semantic and syntactic processing to our feature-based TRF models. For this, we have employed the surprisal metric based on information theory (73). Surprisal has been successfully used to quantify semantic and syntactic processing costs on a word-by-word basis and has been successfully used to capture semantic and syntactic processing from EEG recordings of naturalistic speech in recent years (e.g., 74,75). In line with recent studies, we have computed lexical-semantic information for each word using word form surprisal from GPT-2. Given the big disagreement about the appropriate computational modeling of syntactic structure in the literature, syntactic information was quantified as part-of-speech surprisal, calculated from a Universal Dependencies parse. In addition, we employed word frequency as a proxy for age of acquisition (76) Inclusion of word frequency, semantic and syntactic surprisal into the TRF models did only change the results for phonological feature acquisition quantitatively, but did not affect the main outcomes and conclusions qualitatively.

However, this finding does not exclude the possibility of an interaction between higher order linguistic processing and phonological feature processing. As a next step, we have therefore assessed how the feature-based prediction accuracy in the native language is moderated by either word frequency, word expectancy, or part-of-speech expectancy. We performed a median split on word frequency, GPT-2 surprisal, and part-of-speech surprisal respectively, of all content words. Given the generally high frequency of function words and the higher impact of word frequency on content words, which already arises in infancy (77), we have limited this analysis to content words. We performed a median split of frequency, GPT-2 surprisal, and part-of-speech surprisal and computed the prediction accuracy separately for high and low frequency, GPT-2 surprisal, and part-of-speech surprisal for every fold of the cross-validation procedure; we then averaged prediction values within high/low, across folds. We then conducted separate mixed-effects models for word frequency (high vs. low), GPT-2 surprisal and part-of-speech surprisal values (high vs. low). The model for word frequency showed no main effect, $t = -0.81$, $p = .421$, nor an interaction between word frequency and age, $t= 1.71$, $p = .092$. The surprisal models also showed no significant effects of word surprisal on the prediction accuracy for its features (GPT-2: main effect: $t = -0.2$, $p = .841$; surprisal x age: $t = 1.04$, $p = .304$; part-of-speech: main effect: $t = 0.22$, $p = .829$; surprisal x age: $t = -0,3$, $p = .764$). We therefore find no evidence that higher-order linguistic processing influenced phonological feature processing in this study.

Supplementary Figure S1

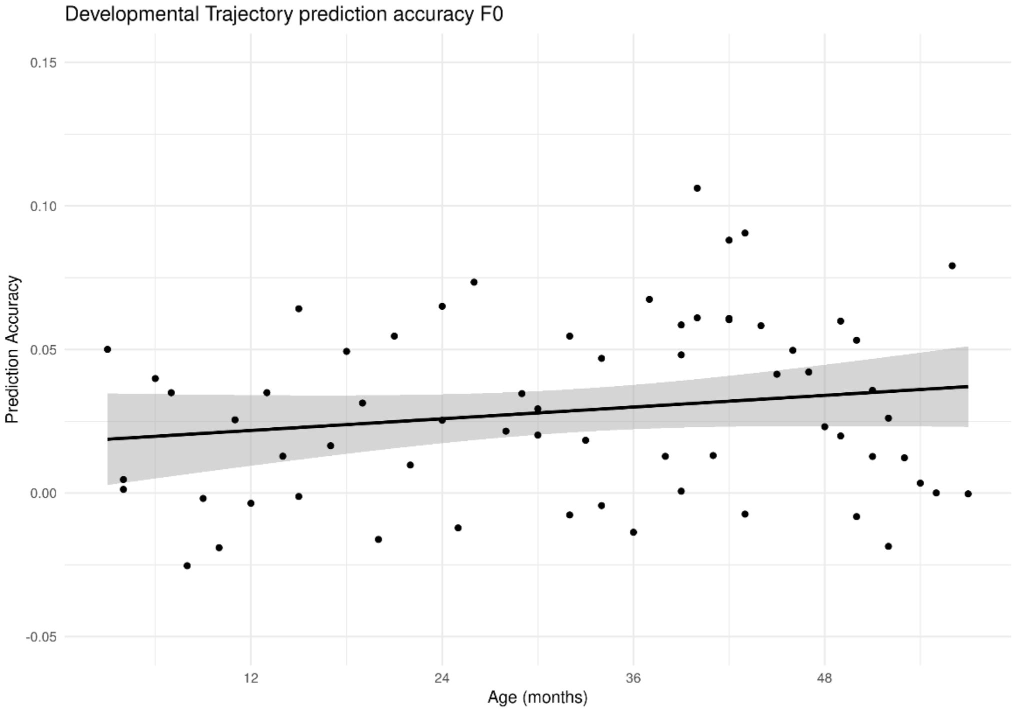

Figure S1: Development of prediction accuracy to F0 across age. Development of prediction accuracy to F0 across age. Dots indicate the observed prediction accuracy minus a baseline value obtained from a permutation of time-shifted F0 values. F0 processing significantly increases with age. F0 processing is significantly above baseline for the youngest age in our sample (i.e., 3 months), indicating that prosodic processing abilities are established early.

REFERENCES AND NOTES

1. P. K. Kuhl, Early language acquisition: Cracking the speech code. *Nat. Rev. Neurosci.* **5**, 831–843 (2004).

2. J. F. Werker, R. C. Tees, Cross-language speech perception: Evidence for perceptual reorganization during the first year of life. *Infant Behav. Dev.* **7**, 49–63 (1984).

3. F.-M. Tsao, H.-M. Liu, P. K. Kuhl, Perception of native and non-native affricate-fricative contrasts: Cross-language tests on adults and infants. *J. Acoust. Soc. Am.* **120**, 2285–2294 (2006).

4. S. Tsuji, A. Cristia, Perceptual attunement in vowels: A meta-analysis. *Dev. Psychobiol.* **56**, 179–191 (2014).

5. K. Zacharaki, N. Sebastian-Galles, Before perceptual narrowing: The emergence of the native sounds of language. *Inf. Dent.* **27**, 900–915 (2022).

6. S. Ortiz-Mantilla, J. A. Hämäläinen, T. Realpe-Bonilla, A. A. Benasich, Oscillatory dynamics underlying perceptual narrowing of native phoneme mapping from 6 to 12 months of age. *J. Neurosci.* **36**, 12095–12105 (2016).

7. A. N. Bosseler, S. Taulu, E. Pihko, J. P. Mäkelä, T. Imada, A. Ahonen, P. K. Kuhl, Theta brain rhythms index perceptual narrowing in infant speech perception. *Front. Psychol.* **4**, 690 (2013).

8. A. Lahiri, H. Reetz, Distinctive features: Phonological underspecification in representation and processing. *J. Phon.* **38**, 44–59 (2010).

9. N. Mesgarani, C. Cheung, K. Johnson, E. F. Chang, Phonetic feature encoding in human superior temporal gyrus. *Science* **343**, 1006–1010 (2014).

10. N. Mesgarani, S. V. David, J. B. Fritz, S. A. Shamma, Phoneme representation and classification in primary auditory cortex. *J. Acoust. Soc. Am.* **123**, 899–909 (2008).

11. G. Gennari, S. Marti, M. Palu, A. Fló, G. Dehaene-Lambertz, Orthogonal neural codes for speech in the infant brain. *Proc. Natl. Acad. Sci. U.S.A.* **118**, e2020410118 (2021).

12. V. Leong, U. Goswami, Acoustic-emergent phonology in the amplitude envelope of child-directed speech. *PLOS ONE* **10**, e0144411 (2015).

13. A. A. Benasich, P. Tallal, Infant discrimination of rapid auditory cues predicts later language impairment. *Behav. Brain Res.* **136**, 31–49 (2002).

14. K. H. Menn, C. Männel, L. Meyer, Does electrophysiological maturation shape language acquisition? *Perspect. Psychol. Sci.* (2023); https://doi.org/10.1177/17456916231151584.

15. G. M. Di Liberto, J. A. O'sullivan, E. C. Lalor, Low-frequency cortical entrainment to speech reflects phoneme-level processing. *Curr. Biol.* **25**, 2457–2465 (2015).

16. N. Schaworonkow, B. Voytek, Longitudinal changes in aperiodic and periodic activity in electrophysiological recordings in the first seven months of life. *Dev. Cognit. Neurosci.* **47**, 100895 (2021).

17. R. Pivik, A. Andres, K. B. Tennal, Y. Gu, H. Downs, B. J. Bellando, K. Jarratt, M. A. Cleves, T. M. Badger, Resting gamma power during the postnatal critical period for GABAergic system development is modulated by infant diet and sex. *Int. J. Psychophysiol.* **135**, 73–94 (2019).

18. D. Cellier, J. Riddle, I. Petersen, K. Hwang, The development of theta and alpha neural oscillations from ages 3 to 24 years. *Dev. Cognit. Neurosci.* **50**, 100969 (2021).

19. J. C. Ziegler, U. Goswami, Reading acquisition, developmental dyslexia, and skilled Reading across languages: A psycholinguistic grain size theory. *Psychol. Bull.* **131**, 3–29 (2005).

20. R. Gess, C. Lyche, T. Meisenburg, *Phonological Variation in French: Illustrations from Three Continents*, vol. 11 (John Benjamins Publishing, 2012).

21. R. Wiese, *Phonetik und Phonologie* (utb GmbH, 2010).

22. A. V. Fox, Evidence from german-speaking children, in *Phonological Development and Disorders in Children: A Multilingual Perspective*, H. Zhu, B. Dodd, Eds. (2006), pp. 56–80.

23. A. A. MacLeod, A. Sutton, N. Trudeau, E. Thordardottir, The acquisition of consonants in québécois french: A cross-sectional study of pre-school aged children. *Int. J. Speech Lang. Pathol.* **13**, 93–109 (2011).

24. M. Havy, C. Bouchon, T. Nazzi, Phonetic processing when learning words: The case of bilingual infants. *Int. J. Behav. Dev.* **40**, 41–52 (2016).

25. K. L. Pike, *The Intonation of American English* (University of Michigan Press, 1945).

26. D. Abercrombie, *Elements of General Phonetics* (Edinburgh University Press, 1967).

27. C. Féry, *Intonation and Prosodic Structure* (Cambridge Univ. Press, 2017).

28. J. Mehler, P. Jusczyk, G. Lambertz, N. Halsted, J. Bertoncini, C. Amiel-Tison, A precursor of language acquisition in young infants. *Cognition* **29**, 143–178 (1988).

29. E. Partanen, S. Pakarinen, T. Kujala, M. Huotilainen, Infants' brain responses for speech sound changes in fast multifeature mmn paradigm. *Clin. Neurophysiol.* **124**, 1578–1585 (2013).

30. A. J. Anderson, S. Perone, Developmental change in the resting state electroencephalogram: Insights into cognition and the brain. *Brain Cogn.* **126**, 40–52 (2018).

31. G. Xu, K. G. Broadbelt, R. L. Haynes, R. D. Folkerth, N. S. Borenstein, R. A. Belliveau, F. L. Trachtenberg, J. J. Volpe, H. C. Kinney, Late development of the GABAergic system in the human cerebral cortex and white matter. *J. Neuropathol. Exp. Neurol.* **70**, 841–858 (2011).

32. T. Nazzi, C. Floccia, J. Bertoncini, Discrimination of pitch contours by neonates. *Infant Behav. Dev.* **21**, 779–784 (1998).

33. J. Gervain, The role of prenatal experience in language development. *Curr. Opin. Behav. Sci.* **21**, 62–67 (2018).

34. T. Nazzi, F. Ramus, Perception and acquisition of linguistic rhythm by infants. *Speech Commun.* **41**, 233–243 (2003).

35. L. Frazier, K. Carlson, C. Clifton Jr., Prosodic phrasing is central to language comprehension. *Trends Cogn. Sci.* **10**, 244–249 (2006).

36. K. H. Menn, C. Michel, L. Meyer, S. Hoehl, C. Männel, Natural infant-directed speech facilitates neural tracking of prosody. *Neuroimage* **251**, 118991 (2022).

37. A. Attaheri, Á. N. Choisdealbha, G. M. Di Liberto, S. Rocha, P. Brusini, N. Mead, H. Olawole-Scott, P. Boutris, S. Gibbon, I. Williams, C. Grey, S. Flanagan, U. Goswami, Delta- and theta-band cortical tracking and phase-amplitude coupling to sung speech by infants. *Neuroimage* **247**, 118698 (2022).

38. L. R. Gleitman, E. Wanner, *Language Acquisition: The State of the Art* (CUP Archive, 1982).

39. K. Hirsh-Pasek, D. G. K. Nelson, P. W. Jusczyk, K. W. Cassidy, B. Druss, L. Kennedy, Clauses are perceptual units for young infants. *Cognition* **26**, 269–286 (1987).

40. M. Soderstrom, A. Seidl, D. G. K. Nelson, P. W. Jusczyk, The prosodic bootstrapping of phrases: Evidence from prelinguistic infants. *J. Mem. Lang.* **49**, 249–267 (2003).

41. J. Gervain, A. Christophe, R. Mazuka, Prosodic bootstrapping, in *The Oxford Handbook of Language Prosody*, C. Gussenhoven, A. Chen (Oxford Univ. Press, 2020), pp. 563–574.

42. D. Crystal, *A Dictionary of Linguistics and Phonetics. Updated and Enlarged* (Basil Blackwell, 1997).

43. A. Cutler, D. Dahan, W. Van Donselaar, Prosody in the comprehension of spoken language: A literature review. *Lang. Speech* **40**, 141–201 (1997).

44. K. H. Menn, E. Ward, R. Braukmann, C. van den Boomen, J. Buitelaar, S. Hunnius, T. M. Snijders, Neural tracking in infancy predicts language development in children with and

without family history of autism. *Neurobiol. Lang.* **3**, 495–514 (2022).

45. U. Goswami, Speech rhythm and language acquisition: An amplitude modulation phase hierarchy perspective. *Ann. N. Y. Acad. Sci.* **1453**, 67–78 (2019).

46. V. Leong, M. Kalashnikova, D. Burnham, U. Goswami, The temporal modulation structure of infant-directed speech. *Open Mind.* **1**, 78–90 (2017).

47. K. M. Hartman, N. B. Ratner, R. S. Newman, Infant-directed speech (ids) vowel clarity and child language outcomes. *J. Child Lang.* **44**, 1140–1162 (2017).

48. D. Raneri, K. Von Holzen, R. Newman, N. B. Ratner, Change in maternal speech rate to preverbal infants over the first two years of life. *J. Child Lang.* **47**, 1263–1275 (2020).

49. C. Bergmann, S. Tsuji, A. Cristia, *Interspeech 2017* (2017), pp. 2103–2107.

50. F. Ramus, M. D. Hauser, C. Miller, D. Morris, J. Mehler, Language discrimination by human newborns and by cotton-top tamarin monkeys. *Science* **288**, 349–351 (2000).

51. F. Schiel, Automatic phonetic transcription of non-prompted speech (1999).

52. T. Kisler, U. Reichel, F. Schiel, Multilingual processing of speech via web services. *Comput. Speech Lang.* **45**, 326–347 (2017).

53. P. Boersma, Praat, a system for doing phonetics by computer. *Glot. Int.* **5**, 341–345 (2001).

54. T. Schmidt, The database for spoken German-DGD2. *Proceedings of the Ninth conferenceon International Language Resources and Evaluation (LREC'14)*, Reykjavik, Iceland [European Language Resources Association (ELRA), 2014], pp. 1451–1457.

55. A. Delorme, S. Makeig, Eeglab: An open source toolbox for analysis of single-trial eeg dynamics including independent component analysis. *J. Neurosci. Methods* **134**, 9–21 (2004).

56. R. Oostenveld, P. Fries, E. Maris, J.-M. Schoffelen, Fieldtrip: Open source software for advanced analysis of meg, eeg, and invasive electrophysiological data. *Comput. Intell.*

NeuRosci. **2011**, 156869 (2011).

57. K. Lopez, A. Monachino, S. Morales, S. Leach, M. Bowers, L. Gabard-Durnam, Happilee: Happe in low electrode electroencephalography, a standardized pre-processing software for lower density recordings. *Neuroimage* **260**, 119390 (2022).

58. L. J. Gabard-Durnam, A. S. Mendez Leal, C. L. Wilkinson, A. R. Levin, The harvard automated processing pipeline for electroencephalography (happe): Standardized processing software for developmental and high-artifact data. *Front. Neurosci.* **12**, 97 (2018).

59. F. Perrin, J. Pernier, O. Bertrand, J. F. Echallier, Spherical splines for scalp potential and current density mapping. *Electroencephalogr. Clin. Neurophysiol.* **72**, 184–187 (1989).

60. S. Jessen, J. Obleser, S. Tune, Neural tracking in infants–An analytical tool for multisensory social processing in development. *Dev. Cogn. Neurosci.* **52**, 101034 (2021).

61. M. J. Crosse, G. M. Di Liberto, A. Bednar, E. C. Lalor, The multivariate temporal response function (mtrf) toolbox: A matlab toolbox for relating neural signals to continuous stimuli. *Front. Hum. Neurosci.* **10**, 604 (2016).

62. G. M. Di Liberto, J. Nie, J. Yeaton, B. Khalighinejad, S. A. Shamma, N. Mesgarani, Neural representation of linguistic feature hierarchy reflects second-language proficiency. *Neuroimage* **227**, 117586 (2021).

63. G. Dehaene-Lambertz, S. Baillet, A phonological representation in the infant brain. *Neuroreport* **9**, 1885–1888 (1998).

64. G. P. Háden, R. Németh, M. Török, I. Winkler, Mismatch response (mmr) in neonates: Beyond refractoriness. *Biol. Psychol.* **117**, 26–31 (2016).

65. M. Cheour, K. Alho, R. Ceponiené, K. Reinikainen, K. Sainio, M. Pohjavuori, O. Aaltonen, R. Näätänen, Maturation of mismatch negativity in infants. *Int. J. Psychophysiol.* **29**, 217–226 (1998).

66. A. Keitel, R. A. Ince, J. Gross, C. Kayser, Auditory cortical delta-entrainment interacts with oscillatory power in multiple fronto-parietal networks. *Neuroimage* **147**, 32–42 (2017).

67. D. Bates, M. Mächler, B. Bolker, S. Walker, Fitting linear mixed-effects models using lme4. *J. Stat. Softw.* **67**, 1–48 (2015).

68. R Core Team, *R: A Language and Environment for Statistical Computing* (R Foundation for Statistical Computing, 2017).

69. RStudio Team, *RStudio: Integrated Development Environment for R*, (RStudio, PBC, 2020).

70. A. Kuznetsova, P. B. Brockhoff, R. H. B. Christensen, lmerTest package: Tests in linear mixed effects models. *J. Stat. Softw.* **82**, 1–26 (2017).

71. A. D. Friederici, A. Pannekamp, C.-J. Partsch, U. Ulmen, K. Oehler, R. Schmutzler, V. Hesse, Sex hormone testosterone affects language organization in the infant brain. *Neuroreport* **19**, 283–286 (2008).

72. N. Chomsky, M. Halle, *The Sound Pattern of English* (Harper & Row, Publishers, 1968).

73. J. Hale, A probabilistic Earley parser as a psycholinguistic model, in *Second Meeting of the North American Chapter of the Association for Computational Linguistics* (2001).

74. M. Heilbron, K. Armeni, J. M. Schoffelen, P. Hagoort, F. P. De Lange A hierarchy of linguistic predictions during natural language comprehension. *Proc. Natl. Acad. Sci. U.S.A.* **119**, e2201968119 (2022).

75. J. R. Brennan, J. T. Hale, Hierarchical structure guides rapid linguistic predictions during naturalistic listening. *PLOS ONE* **14**, e0207741 (2019).

76. B. Ambridge, E. Kidd, C. F. Rowland, A. L. Theakston, The ubiquity of frequency effects in first language acquisition. *J. Child Lang.* **42**, 239–273 (2015).

77. B. Höhle, J. Weissenborn, German-learning infants' ability to detect unstressed closed-class elements in continuous speech. *Dev. Sci.* **6**, 122–127 (2003).
